# Supplementary material for: Histamine-2 receptor antagonist famotidine modulates cardiac stem cell characteristics in hypertensive heart disease
Source: PeerJ. 2017 Oct 9;5:e3882. doi: 10.7717/peerj.3882 (PMC5637875; doi:10.7717/peerj.3882)
Supplement: Supplemental Information 1 [file peerj-05-3882-s001.pdf]

	WISTAR						
							growth kin[illegible]
	*104				mean	sd	
	0	1	1	1	1	0	0
	2	19	23	25	22.33333	3.05505	2
	4	29	41	50	40	10.53565	4
	6	56	65	71	64	7.549834	6
	8	75	86	98	86.33333	11.50362	8
	10	126	139	113	126	13	10
PDT		1.433	1.405	1.466	1.434667		
GR		0.494	0.473	0.484	0.483667		
	SHR CONTROL						
	*104						
	0	1	1	1	1	0	
	2	14	20	15	16.33333	3.21455	
	4	22	19	25	22	3	
	6	27	35	30	30.66667	4.041452	mean
	8	42	51	39	44	6.244998	sd
	10	71	67	78	72	5.567764	
pdt		1.63	1.65	1.59	1.623333	0.030551	
gr		0.426	0.421	0.436	0.427667	0.007638	
	SHR Treated						
	*104						
	0	1	1	1	1	0	
	2	15	22	11	16	5.567764	
	4	29	33	44	35.33333	7.767453	
	6	46	52	39	45.66667	6.506407	
	8	79	66	81	75.33333	8.144528	
	10	111	121	107	113	7.211103	
pdt		1.47	1.45	1.48	1.466667	0.015275	
gr		0.471	0.48	0.467	0.472667	0.006658	
							mean
							sd

CFU

	WST	SHR	Treated
	99	45	77
	87	62	88
	82	59	69
mean	89.33333333	55.33333333	78
sd	8.736894948	9.073771726	9.539392014

CFU

WST	89.33333
SHR	55.33333
Treated	78

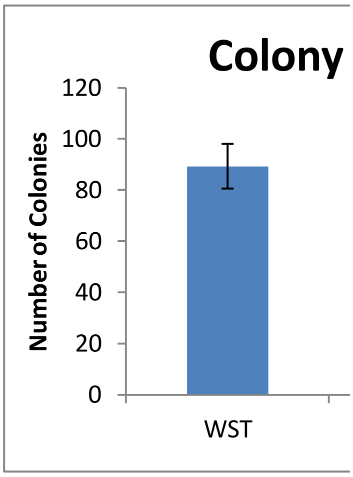

Senescent assay

	WST	SHR	Treated
	1.5	3.5	1.8
	0.6	2.6	0.9
	1.2	3	2
mean	1.1	3.033333333	1.566666667
sd	0.458257569	0.450924975	0.585946528

Senescent assay

WST	1.1
SHR	3.033333
Treated	1.566667

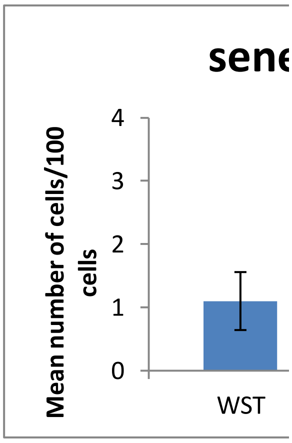

Migration Assay

	WST	SHR	Treated
	79	43	71
	87	49	59
	62	51	66
mean	76	47.66666667	65.33333333
sd	12.76714533	4.163331999	6.027713773

WST	76
SHR	47.66667
Treated	65.33333

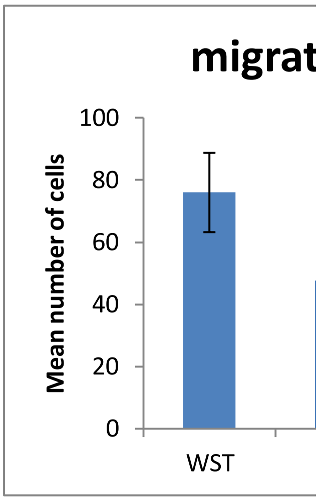

DCFDA

WST	145.3333
SHR	224.3333
Treated	171

DCFDA

	WST	SHR	Treated
	134	231	172
	157	243	156
	145	199	160
mean	145.3333333	224.3333333	162.6666667
sd	11.50362262	22.74496281	8.326663998

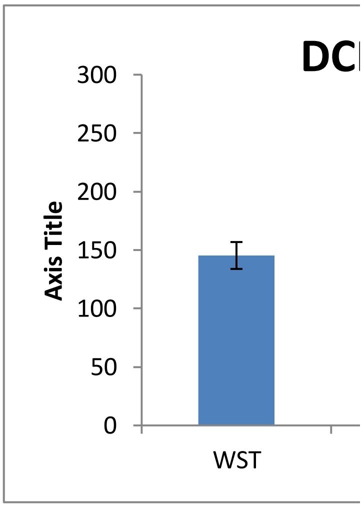

etics

WST	SHR	Treated
1	1	1
22.33333	16.33333	16
40	22	35.33333333
64	30.66667	45.66666667
86.33333	44	75.33333333
126	72	113

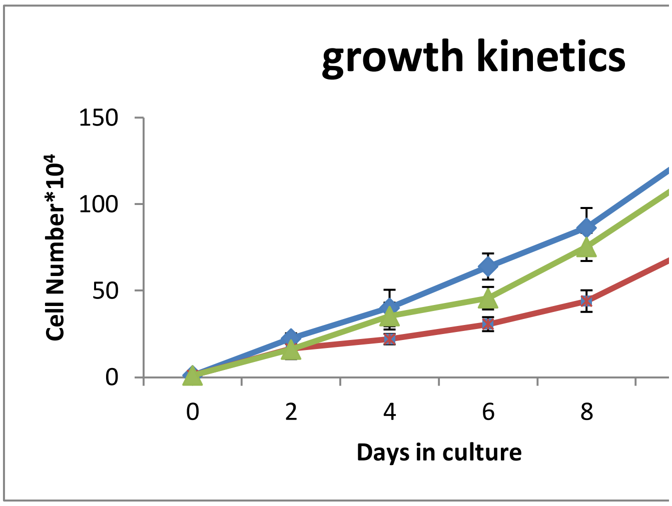

pdt

wst	shr	Treated				t test
1.433	1.63	1.47	PDT			wst/shr
1.405	1.65	1.45	WST	1.434667	0.030199	shr/treated
1.466	1.59	1.48	SHR	1.623333	0.039501	wst/treated
1.434667	1.623333	1.466666667	Treated	1.466667	0.025534	
0.030534	0.030551	0.015275252				ANOVA p<0.01

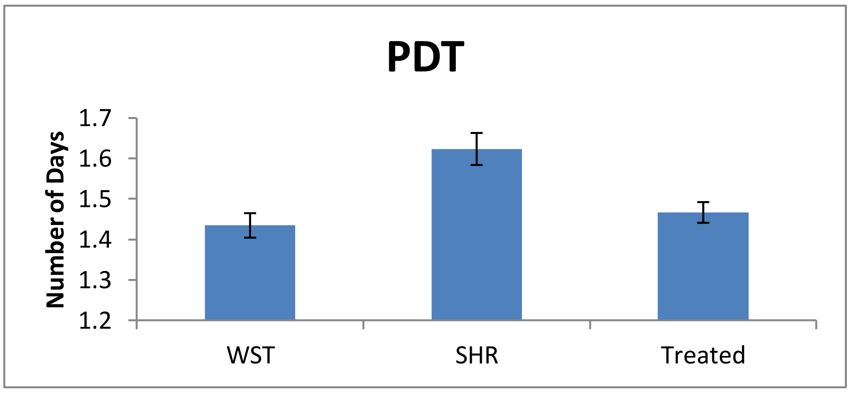

gr

wst	shr	Tempol	gr			t test
0.494	0.426	0.471	WST	0.483667	0.010504	wst/shr
0.473	0.421	0.48	SHR	0.427667	0.007638	shr/treated
0.484	0.436	0.467	Treated	0.472667	0.006658	wst/treated
0.483667	0.427667	0.472666667				
0.010504	0.007638	0.006658328				ANOVA p<0.01

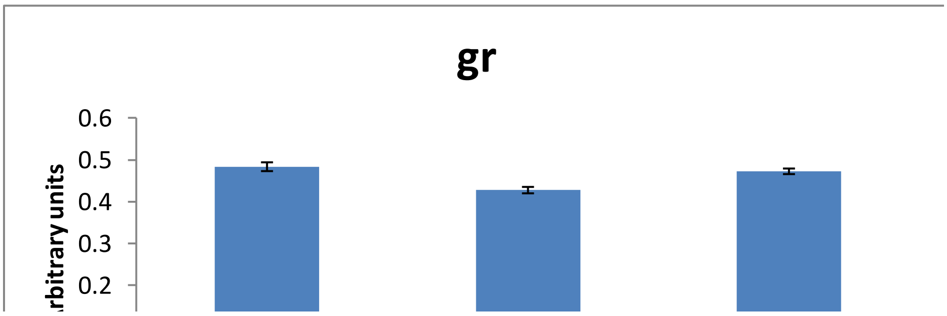

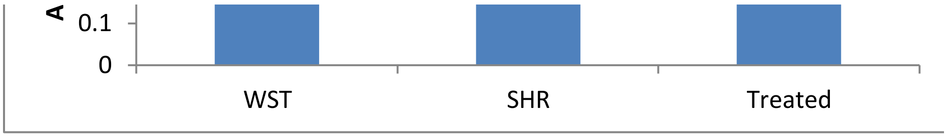

CFU

t test

8.736895	wst/shr	0.009481789	**
9.073772	shr/treated	0.040658463	#
9.539392	wst/treated	0.203745471	

ANOVAp<0.01

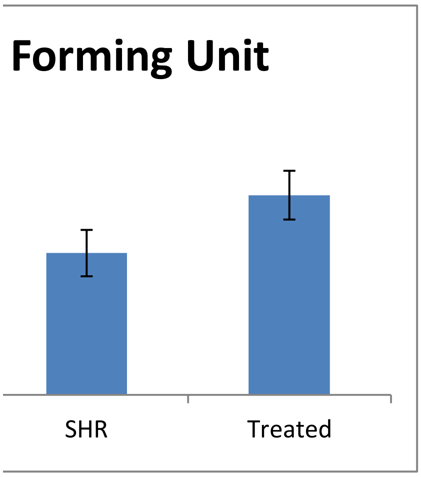

Senescent assay

t test

0.458258	wst/shr	0.006478	**
0.450925	shr/treated	0.026394	#
0.585947	wst/treated	0.338307	

ANOVAp<0.01

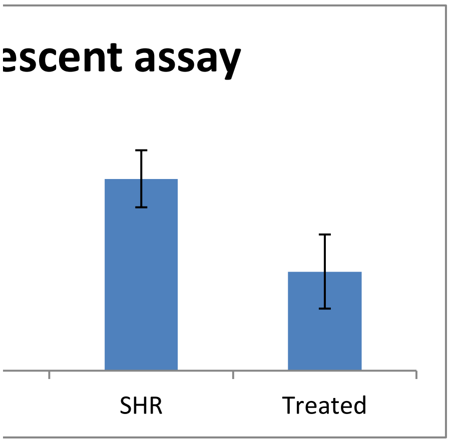

Migration Assay

t test

12.76715	wst/shr	0.021686432	*
4.163332	shr/treated	0.013952224	#
6.027714	wst/treated	0.260799105	

ANOVAp<0.01

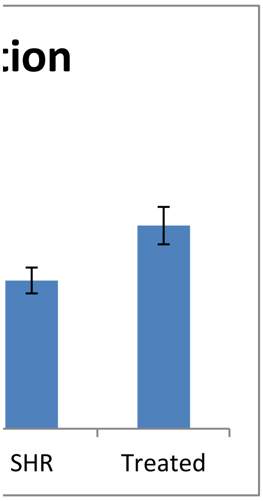

DCFDA

t test

11.50362	wst/shr	0.005813654	**
22.74496	shr/treated	0.01160274	#
9.539392	wst/treated	0.102017814	

ANOVAp<0.01

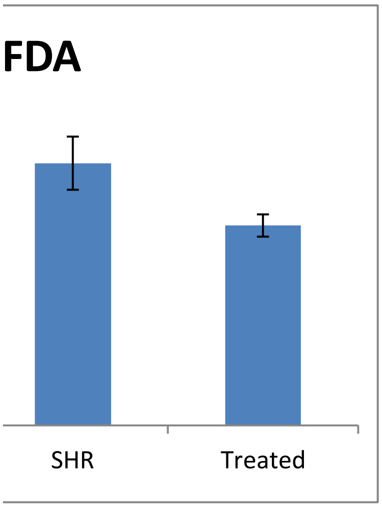

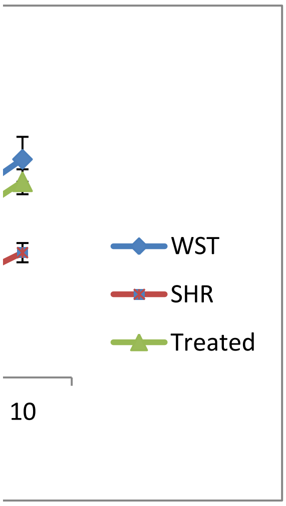

0.001636 **
0.001359 ##
0.179828

0.001718 **
0.001536 ##
0.200288
